# Combination of Probiotics and Natural Compounds to Treat Multiple Sclerosis via Warburg Effect

**DOI:** 10.34172/apb.2022.057

**Published:** 2021-09-29

**Authors:** Anjali Kandiruthi Ravi, Saradhadevi Kuppusami Muthukrishnan

**Affiliations:** Department of Biochemistry, Bharathiar University, Coimbatore, India.

**Keywords:** Medicinal plants, Multiple sclerosis, Prebiotics, Probiotics, Warburg effect

## Abstract

Multiple sclerosis (MS) is a chronic demyelinating disease of the central nervous system (CNS). It is an auto-immune disorder. Its usual symptoms are unique to each person. In MS lesions vast fractions of pyruvate molecules are instantly transformed into lactate. This reprogramming mechanism of glycolysis is known as the Warburg effect. MS has no efficient treatment yet. Hence, there is a requirement for profitable immunomodulatory agents in MS. Probiotics perform as an immunomodulator because they regulate the host’s immune responses. Its efficacy gets enhanced for an extended period when it combines with prebiotics. In this review, we focus on the metabolic alterations behind the MS lesions via the Warburg effect, and also suggesting, the combined efficacy of prebiotics and probiotics for the effective treatment of MS without side effects. The Warburg effect mechanism intensifies the infiltration of activated T-cells and B-cells into the CNS. It provokes the inflammation process on the myelin sheath. The infiltration of immune cells can be inhibited by the combination therapy of probiotics and prebiotics. By this review, we can recommend that the idea of this combinational therapy can do miracles in the treatment of MS in the future.

## Introduction

Multiple sclerosis (MS) is an auto-immune disorder, in which our immune system attacks our healthy myelin sheath in the brain, spinal cord and the optic nerves get degraded. It alters the signal transduction of the brain. Approximately, 2.5 million people are typically affected by MS worldwide.^1^ Epidemiologic researches have revealed precisely that females are more affected by MS than males.^2^ MS arises with relapsing-remitting MS and drives to a chronic neuro-degenerative condition, known as primary and secondary progressive MS.^3^ Due to the unique characteristics of MS, its symptoms are diverse. 80% of MS patients experience fatigue, unusual or excessive whole-body tiredness. It can severely affect a person’s fundamental quality of successful life. This prominently includes the somatic symptoms such as motility difficulties, fatigue, weakness, visual dilemmas, reproductive problems, psychological symptoms such as depression, anxiety, mental issues and neuro-cognitive symptoms such as: lack of attentiveness, memory, language and processing speed.^4^ The pathology of MS include various factors.^5^ The activated cytotoxic T cells infiltrate into the blood-brain barrier (BBB) and react defensively against the myelin sheath due to the activation of microglia and macrophages.^6^ When the myelin steadily and continuously gets destroyed, nerve signals become moderate or may even cease, which prompt neurological problems. BBB is designed by a specific endothelial cell without membrane pores which are sealed with tight junctions.^7^ In MS, the stimulated leukocytes can enhance the membrane permeability of the BBB by the expression and secretion of inflammatory cytokines, soluble constituents, reactive oxygen species and matrix metalloproteinase.^8^ Warburg effect plays a chief role in demyelination and disease progression. Wnt-signaling pathway (especially β-catenin, Wnt3a and APC protein expression),^9^ JAK/STAT signaling pathway,^10^ NF-kB signaling pathway^11^ and PImT3K/Akt/mTOR pathway^12^ are highly expressed in MS lesions.

Different medications are available for the treatment of MS which includes: orals (fingolimod, teriflunomide and dimethyl fumarate), injectable-(interferons, glatiramer acetate and mitoxantrone), monoclonal antibodies (natalizumab, alemtuzumab, daclizumab and ocrelizumab).^13^ Although, certain medicines are not safe in the long-term as they cause severe side effects. Herbal therapies and probiotic supplements seem to be more efficient in the treatment of MS. The medicinal plants reduce neuronal inflammation and improve the quality of sleep, ease muscle stiffness and reduce bladder trouble.^14^ Probiotics in turn, equally possess significant functions in defeating autoimmune disorder and gut dysbiosis.^15^ Hence, the use of sufficient Probiotics and herbal medicine can reduce the inflammation in the central nervous system (CNS) without any severe side effects. Phytochemicals in the plants act as prebiotics. In this review, we seek to discuss the possibilities of combination therapy using probiotics and medicinal plants as prebiotics for the treatment of MS.

## Warburg effect in multiple sclerosis

The human brain uses one half of all the glucose in the body for the growth of nerve cells. Glucose molecules are delivered to astrocytes and oligodendrocytes by glucose transport protein (Glut-1). If the glucose levels get reduced, the signal transduction will be interrupted. Oligodendrocytes, the myelin precursor cells, oxidize glucose into lactate through a lactate shuttle system. It is then converted into pyruvate by the enzyme lactate dehydrogenase. Later, pyruvate molecules get oxidized in mitochondria by oxidative phosphorylation for the synthesis of high-energy ATP molecules.^16^ However, in MS cells, an enormous volume of cytosolic pyruvate is transformed into lactate, even in the presence of sufficient oxygen. This metabolic shift is defined as the Warburg effect.^17^ Aerobic glycolysis produce two molecules of ATP per cycle when compared to oxidative phosphorylation. Studies reveal that ATP generation per cycle in aerobic glycolysis is extremely quicker than the mitochondrial oxidative phosphorylation and produces more ATP which can be utilized for the activation of T-cells.^18^ Activated T-cells infiltrate into BBB and stimulates the autoimmune mechanism and hence causes the myelin degeneration and axonal destruction.

**Figure 1:**
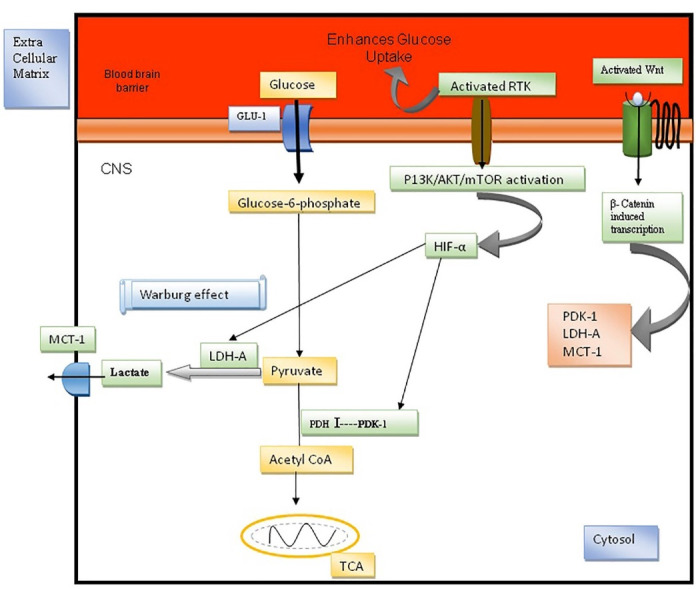
Warburg effect in MS.

The brain occupies 2% of total body weight and utilizes about 20% of the entire glucose and oxygen. Glucose transporters will adequately provide ample glucose molecules to the nerve cells.^19^ Cytosolic pyruvate cannot oxidize further and becomes reduced to lactate under hypoxia.^20^ Hypoxia inducing factor-α (HIF-1α) induces pyruvate dehydrogenase kinase1 for inhibiting the catalytic activity of PDH by phosphorylating it, utilizing active ATP molecule. The activated immune cells stimulates the autoimmune mechanism of the transcription factor NF-κB for the continuous production of pro-inflammatory cytokines, interleukin-1β (IL-1β) and tumor necrosis factor-α (TNF).^21^ These cytokines trigger the metabolic shift from oxidative phosphorylation to aerobic glycolysis.^22^ The membrane-bound receptors TLR2, TLR4 and TLR9 properly promote the glucose transporter GLUT1 for more glucose uptake for the lactate production.^23^ The bulk production of lactate leads to neuronal death and myelin damage in MS.^24^ During the MS condition, the homeostasis of energy metabolism is impaired by mitochondrial dysfunction with limiting oxidative-phosphorylation.^25^ The precipitous rise in lactate molecules, may typically trigger the progression of MS cells. In investigations, it is evident that the lactate levels are increased in MS lesions. It is undoubtedly the reason for mitochondrial dysfunction and neuroinflammation.^26,27^ The Warburg effect mediated demyelinating process in MS is explained in Figure 1.

## Altered immune mechanism via Warburg effect

**Figure 2:**
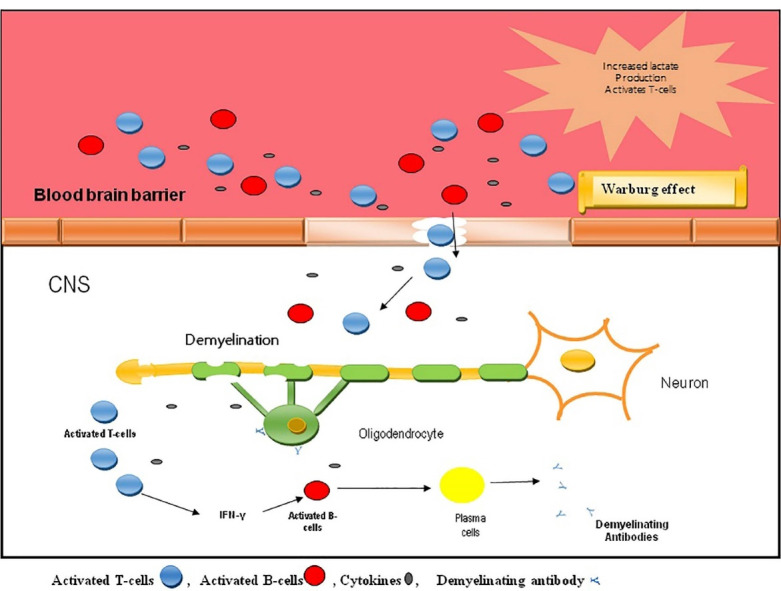
Factors involving demyelination process.^31^

In MS, the immune cells (specialized CD4+T cells and B cells) become stimulated in the peripheral lymph tissues and penetrate into the CNS through BBB. Activated immune cells secrete cytokines to induce inflammation in CNS. The stimulated immune cells precisely require more energy in the form of ATP. For getting sufficient quantities of energy, T-cells modify their energy metabolism through Warburg effect which instantly follows aerobic glycolysis for the rapid production of ATP and other metabolic intermediates.^23^ Activated T-cells in MS convert pyruvate into lactate.^28^ Normal T-cells generate ATP through catabolism of glucose, amino acids and lipids and mainly oxidative phosphorylation. But the activated T cells shift the glucose metabolism to aerobic glycolysis (Warburg effect) for energy production.^29^ Immune activated T cells are divided into several subunits which includes cell-mediated immune response (specialized Th1 cells), humoral immunity (specialized Th2 cells), active inflammation (specialized Th17 cells) and regulatory T cells. Specialized Th17 cells participate in neuroinflammation and secrete cytokine IL-17.^30^ Bulk production of lactate provoke the gene modification of cytotoxic T-cells as illustrated in Figure 2. Pro-inflammatory mediators, such as cytokines, interleukins (IL-6, IL-17, IL-22), TNF-α are synthesized by T-cells.

Each stimulated B-cell acts as the antigen-presenting cells and later they will be transformed into plasmacytes for the secretion of demyelinating antibodies. Gradual infiltration of B cells and specific T cells into the CNS provoke the immune response of TLR receptors.^32^ These receptors, TLR2 and TLR4 enhance the functional differentiation of T-cells into Th-1. Specialized Th-17 cells secrete IL1, IL6 and IL12. Th1 and specialized Th17 cells are liable for the secretion of the cytokines Interferon gamma (IFN-γ) and IL17, which leads to the neuronal inflammation.^33^ TLR3 induces NF-kB pathway through the expressed TRIF protein for the secretion of type 1 IFNs.^34^ IFN-β represents a distinct type-1 IFN which is reliably used for the treatment of relapsing-remitting MS, upon the cognitive stimulation of innate immune responses.^35^

## Signaling pathways in MS

During MS the Wnt/Catenin, JAK/STAT, NF-kB and PI3/AKT/mTOR signaling pathways are over expressed. Wnt signaling pathway inhibits the pyruvate oxidation by delivering the factors such as PDK1, Myc gene and the lactate transporter MCT-1.^15^ Wnt/Catenin and PI3/AKT/mTOR pathways elevate the glucose uptake for sustaining the aerobic glycolysis.^17^ The stimulated PI3/AKT/mTOR pathway induces HIF-1α, which defeats oxidative phosphorylation.^13^ PPARγ, a transcription factor, which regulates glucose metabolism and cellular homeostasis. WNT ligands belong to the family of glycoproteins concurring in the chief regulator of the cell cycle, cell regulation and embryogenesis.^36^ Altered PPAR^37^ and WNT/catenin^38^ signaling pathways trigger demyelination through the Warburg effect.^39^ The JAK/STAT pathway is important for the potential development of both adaptive and innate immunity.^40,41^ This specific pathway, abnormally expressed in MS, particularly STAT3 and STAT4 which releases cytokines for the extensive development of lesions on myelin sheath.^42-45^ IL-1β and TNF-α elevates the optimum levels of NF-kB in MS.^46,47^ INF-γ is one of the major pro-inflammatory cytokines observed in MS lesions secreted by T cells, NK cells and macrophages.^48^ By targeting the NF-kB along with its novel inhibitors, it can diminish the pro-inflammatory T-cell responses and thus resist MS.^49^

Enhanced levels of PI3/AKT/mTOR pathway remains a prominent sign of adaptive auto-immunity because it regulates the T-cell activation, proliferation, and apoptosis.^50,51^ The apparent magnitude of mTOR immune activation is directly proportional to the communication between immune T cells and dendritic cells.^52^ The enhanced mTOR signaling is observed only in the initial states of oligodendrocytes formation and not during the maturation phases.^53^ Hence, targeting the PI3K/mTOR pathway might not represent a beneficial strategy for the remyelination method, however, it could slow down/ reduce the MS progression.^12^ These signaling pathways are summarized in Table 1.

**Table 1:** Role of different Signaling pathways in MS

**Signaling pathway**	**Action**	**Reference**
WNT/Catenin pathway	Release of cytokines by CD4^+^ Th17 cells.	^36^
JAK/STAT	Functioning and development of both adaptive and innate immunity.	^41^
PI3/AKT/mTOR	T-cell activation, proliferation, metabolism and apoptosis.	^51^
NF-kB	Maturation of immune cells and production of inflammatory mediators.	^49^

## Use of natural compounds in the treatment of MS

Herbal therapies used for the treatment of MS, are effectively tested in animals and humans.^54^ It is possible to treat the usualsymptoms of MS effectively by practicing herbal medicines which have anti-inflammatory and antioxidant qualities to stop the myelin sheath destruction without side effects. Phytochemicals are the bio-active compounds naturally found in plants and act as prebiotics. Some of the bioactive compounds present in medicinal plants are listed in Table 2.

**Table 2:** Effect of natural compounds in MS

**Bio-active components**	**Study design**	**Type of MS/study models**	**Effect of bioactive component in MS**	**Ref.**
*Curcumin*	Adult female Lewis rats (150-200 g)	Experimental autoimmune encephalomyelitis (EAE) (animal model of MS)	Reduces the oxidative stress Enhances remyelination process	^55^
*Cannabis*	Randomized placebo-controlled, double-blind parallel group study of 160 MS patients	MS	Effective for spasticity associated with MS	^56^
Epigallocatechin-3-gallate	Randomized, double-blind, placebo-controlled, crossover trial of 18 MS patients	Relapsing-remitting MS	Improves muscular metabolism to a greater extent in men than in women	^57^
*Crocus sativus* L.	8-week-old C57BL/6 mice	EAE	Inhibits oxidative stress and prohibits leukocyte infiltration to CNS	^58^
*Ginger*	Female 6- to-8-week age C57BL/6 mice	EAE	Reduces the infiltration of inflammatory cells into the CNS	^59^
*Andrographis paniculata*	Randomized double-blind placebo-controlled trial of 25 patients	Relapsing-remitting MS	Reduces the chronic fatigue	^60^
*Dendropanaxmorbiferus*	13.5 days pregnant females mice or pups	Oligodendrocytes (OLs) primary culture systems	Enhances oligodendrocytes development	^61^
*Terminalia ferdinandiana*	Antibacterial activity against the strains of *A. baylyi* and *P. aeruginosa*	Bacterial triggers of MS	Inhibits the growth of *Acinetobacter baylyi* and *Pseudomonas aeruginosa* which triggers MS	^62^
*Boswellia papyrifera*	Clinical trial of 80 MS patients	Relapsing remitting MS	Improves the visual and spatial memory.	^63^
*Scrophulariamegalantha*	Nineteen C57BL/6 female mice- weighing 18-20g (7-to 9 week-age)	EAE	Down-regulates the production of IFN-γ and IL-17 and up-regulates the anti-inflammatory IL-10	^64^
Saffron	Adult male Wistar rats (200-250 g)	Ethidium bromide induced demyelination	Improves spatial learning memory and antioxidant enzyme activity	^65^
Resveratrol	Male C57Bl/6 mice (20-25 g)	Cuprizone induced demyelination	Improves mitochondrial function, remyelination, motor coordination reduces oxidative stress by enhances the expression of Olig1 gene and inhibited NF-κB signaling pathway	^66^
Resveratrol	Female SJL/J mice (6-week age)	EAE	Exhibits neuro-protective activity by the activation of SIRT1 mechanism	^67^
*Cannabis*	Randomized clinical trial of 144 MS patients	MS	Improves pain relief and muscle stiffness in MS patients	^68^
*Curcumin*	Rat embryonic hippocampal neuron- from 17-days pups	Primary hippocampal neuron cell culture study	Protects axon degradation by inhibiting microglial MyD88/p38 MAPK signaling and nitric oxide production	^69^

Polymerized form of Nano-curcumin reduces the BBB damage, active inflammation and demyelination through enhanced remyelination and reduced oxidative stress.^55^ *Cannabis* extract reduces the pain, and spasticity associated challenges in MS.^56^ Oral administration of epigallocatechin-3-gallate, a naturally derived Catechin of green tea, along with regular exercise improves the muscular metabolism in MS patients.^57^ *Crocus sativus* L. extracts inhibit MS progression by restraining oxidative stress and leukocyte infiltration to CNS.^58^ Ginger extract modulates the expression of IL-27 and IL-33 for the reduced infiltration of inflammatory immune cells into the CNS.^59^ *Andrographis paniculata* minimizes the fatigue associated with MS.^60^ The treatment using *Dendropanaxmorbiferus* leaf extract enhances the oligodendrocyte regeneration in MS patients.^61^ The fruit extracts of *Terminalia ferdinandiana* inhibit the growth of bacterial species which triggers autoimmune response.^62^ Treatment using *Boswellia papyrifera* improves the visual and spatial memory of MS patients.^63^ *Scrophulariamegalantha* extract inhibits the secretion of IFN-γ and IL-17 and increases the formation of IL-10.^64^ The treatment using ethanolic extract of saffronagainst memory loss and oxidative stress, improves the cognitive performance of learning and memory in animal MS models.^65^ The active treatment of MS mice model using resveratrol ameliorates mitochondrial function, reduces oxidative stress, enhances motor co-ordination, activates remyelination through boosting the expression of Olig1 gene and irreversibly inhibits the signaling pathway of NF-κB.^66^ Another study of resveratrolexhibits higher neuro protection and improved mitochondrial function by the activation of novel SIRT1 mechanism and NAD^+^ dependent deacetylase pathway.^67^ *Cannabis* extract shows relief from pain and muscle stiffness in MS patients.^68^ *Curcumin* extracts protects axons from degeneration by inhibiting microglial MyD88/p38 MAPK signaling.^69^

## Probiotics for the treatment of MS

Probiotics comprise vital microbial species which can modulate the immune responses of the host organism healthily by producing antimicrobial agents as bacteriocins.^70^ There are thousands of microbial species in the human gut. Bacteroidetes and Firmicutes are the two principal phyla of healthy gut microbiome. The gut microbiome altered in MS. Probiotics can naturally provoke the anti-inflammatory peripheral immune response in MS patients. Modulating the gut microbiome using probiotics is beneficial to MStreatment.^71^ Some of them are noted in Table 3.

**Table 3:** Probiotics against MS

**Probiotics**	**Study design**	**Type of MS/ study models**	**Effect of probiotics**	**Ref.**
*Lactobacillus, Bifidobacterium* and *Streptococcus*	Clinical trial: MS subjects with glatiramer Acetate treatment - 7 Without treatment - 2 Healthy controls - 13	Relapsing-remitting MS	Inhibits the infiltration of intermediated monocytes into the CNS	^72^
*Lactobacillus paracasei* and * L. plantarum*	Female Lewis rats 6–8-week age	Experimental auto immune myasthenia gravis	Reduces the CNS inflammation Inhibits Th1 and Th17 cytokines	^73^
*Lactobacillus reuteri*	Female mice wild-type (WT) C57BL/6 (10 weeks-age)	EAE	Reduced TH1/TH17 cells and their associated cytokines IFN-g/IL-17	^74^
*Lactobacillus plantarum* and *Bifidobacterium animalis*	Female C57BL/6 mice (8–10 weeks age)	EAE	Improved the state of CD4+CD25+Foxp3+-expressing T-cells in the spleen and the lymph nodes	^75^
*Saccharomyces boulardii*	Double-blind randomized controlled two-group parallel Clinical trial of 50 MS patients	MS	Reduces CNS inflammation, fatigue, pain and oxidative stress	^24^
*Lactobacillus acidophilus, lactobacillus casei, Bifidobacterium bifidum* and *Lactobacillus fermentum*	Randomized, double-blind, placebo-controlled clinical trial of 40 MS patients	MS	Down regulates the gene expressions of IL-8 and TNF-α	^76^
*Lactobacillus casei, Lactobacillus acidophilus, Lactobacillus reuteri, Bifidobacterium bifidum* and *Streptococcus thermophilus*	C57BL/6 mice (6–8 weeks-age)	EAE	MOG-reactive T cell propagation and pro-inflammatory cytokine levels are reduced and improving IL10+ or/and Foxp3+ Treg cells. Inhibits the pro-inflammatory Th1/Th17 polarization.	^77^
*Lactobacillus plantarum* and *Bifidobacterium B94*	32 male Wistar rats	Ethidium bromide induced demyelination	Improves the spatial memory and learning.	^78^
*Lactobacillus acidophilus, Lactobacillus casei, Bifidobacterium bifidum and Lactobacillus fermentum*	Randomized, double-blind, placebo-controlled clinical trial of 60 MS patients	MS	Shows improvements in expanded disability status scale, mental health and HDL- cholesterol levels.	^79^
*Bifidobacterium infantis, Bifidobacterium lactis, Lactobacillus reuteri, Lactobacillus casei, Lactobacillus plantarum and Lactobacillus fermentum*	Randomized, double-blind, placebo-controlled clinical trial of 48 MS patients	MS	Decreases the levels of hsCRP and IL-6 and increased the anti-inflammatory cytokine IL-10	^80^
*Streptococcus thermophilus*	Female SJL/J mice, (6–9 weeks age)	MBP83–99 peptide immunized MS model	Inhibited the secretion of pro-inflammatory cytokines IL-1β and IFN-γ and enhances the secretion of anti-inflammatory cytokines IL-4, IL-5, IL-10	^81^

Administration of VSL3 probiotics mixture comprising of *Lactobacillus, Bifidobacterium* and *Streptococcus* in MS patients, switches their gut microbiota to modulate the anti-inflammatory peripheral innate immune response by regulating the intermediate monocytes.^72^ *Lactobacillus paracasei* and *L. Plantarum* reduces the CNS inflammation by inhibition of pro-inflammatory Th1 and Th17 cytokines in MS.^73^ *Lactobacillus reuteri* mediated treatment of MS changes the gut microbiota and modulates Th1 and Th17 and their associated cytokines.^74^ The combined effect of *Lactobacillus plantarum* A7 and *Bifidobacterium animalis* strains inhibit MS progression by regulating the inflammatory T-cells infiltration into the CNS.^75^ *Saccharomyces boulardii*, a yeast-derived probiotic reduces the CNS inflammation, fatigue, pain and oxidative stress in MS patients.^24^ Oral administration of *Lactobacillus acidophilus, Lactobacillus casei, Bifidobacterium bifidum* and *Lactobacillus fermentum* probiotics inhibits the gene expressions of IL-8 and TNF-α in MS patients.^76^ *Lactobacillus casei, Lactobacillus acidophilus, Lactobacillus reuteri, Bifidobacterium bifidum* and *Streptococcus thermophilus* which slows down the pro-inflammatory Th1/Th17 polarization.^77^

*Lactobacillus plantarum* and *Bifidobacterium* B94 combined treatment promote spatial memory in MS.^78^ Treatment using probiotic capsules which contain *Lactobacillus acidophilus, Lactobacillus casei, Bifidobacterium bifidum* and *Lactobacillus fermentum* shows improvement in mental health and elevated HDL- cholesterol levels.^79^ A clinical trial treatment using probiotics capsule containing *Bifidobacterium infantis, Bifidobacterium lactis, Lactobacillus reuteri, Lactobacillus casei, Lactobacillus plantarum* and *Lactobacillus fermentum* progressively reduced the clinical symptoms and decreased the levels of high‐sensitivity C‐reactive protein and IL-6. Probiotics irreversibly inhibit the inflammatory cytokines and simultaneously increase the anti-inflammatory cytokine IL-10.^80^ Treatment using *Streptococcus thermophilus* as probiotics inhibited the secretion of pro-inflammatory cytokines IL-1β and IFN-γ and also enhanced the secretion of anti-inflammatory cytokines IL-4, IL-5, IL-10 in MS-induced mice model.^81^

## Prebiotics and probiotics in MS

MS is significantly associated with excess inflammation in the brain and spinal cord. Various studies sufficiently revealed, that gut microbiome is altered in MS patients.^24,72,76,79–81^ It is proved that some bio-active compounds produce excessive neuro-protective activity against MS.^56,57,61,64,69^ Prebiotics serve as good food for probiotics. Active inflammation in MS might be due to the profound alterations in the gut microbiome. Recently, various clinical trials were ongoing in this specialized field, and waiting for good results.^82,83^

## Conclusion

The metabolic alterations in MS scientifically proved the significant role of Warburg effect in it. The demyelinating process is started by the immune activation of inflammatory CD4+ and CD8+ lymphocytes cells in the peripheral lymph nodes through the Warburg effect. The stimulated T-cells then differentiate into the Th1, Th17, Th2 and T regulatory cells. Cytotoxic Th1 lymphocytes are responsible for the continuous production of IL-2 and IFN-γ, which triggers cellular immune responses. Th17 lymphocytes trigger active inflammation through the secretion of cytokines IL-17. Cytotoxic Th2 lymphocytes and T regulatory cells secrete IL-4 and IL-10, which mediates humoral immune responses and generate anti-inflammatory cytokines. The activated B cells (antigen-presenting cells) are transformed into plasma cells for the production of demyelinating antibodies. When the immune cells become active, they forcibly displace oxidative phosphorylation to the aerobic glycolysis. Various metabolic pathways (Wnt/catenin, JAK/STAT pathway, NF-kB signaling pathway and direct PI3/AKT/mTOR pathway) are altered in MS.

The enhanced lactate production via aerobic glycolysis may induce T-cell activation. Activated T-cells are the major reason for myelin destruction. The BBB membrane permeability is progressively increased by these activated T-cells. Thus the reactive T-cells and B-cells migrate into the CNS through BBB and trigger the inflammation process on the myelin sheath. The inflammation process easily spreads by gradual infiltration of macrophages and monocytes into the CNS. The myelin attack leads to CNS damage. Gut microbiota can influence the brain. Any alteration in the gut microbiome can indirectly affect the immune system. Thus, probiotics can be used as an immunomodulatory substitute for the treatment of MS. Various studies, convincingly show that anti-inflammatory elements in the medicinal plants naturally have MS healing effect. Consequently, by this review, we can delicately suggest that more researches are needed for identifying the beneficial part of this combinational therapy of probiotics and medicinal plant extracts against MS. Prebiotics and Probiotics treatment can be prominently used as an efficient adjuvant therapy against MS.

## Acknowledgments

The manuscript was not financed from any source.

## Ethical Issues

This work does not contain any studies with animals or human participants conducted by any of the authors.

## Conflict of Interest

The authors declare no conflict of interest in this study.
